# The recent and future PM_2.5_-related health burden in China apportioned by emission source

**DOI:** 10.1038/s44407-025-00006-9

**Published:** 2025-04-28

**Authors:** Jiemei Liu, Jørgen Brandt, Jesper H. Christensen, Zhuyun Ye, Tingsen Chen, Shikui Dong, Camilla Geels, Yuan Yuan, Athanasios Nenes, Ulas Im

**Affiliations:** 1 Key Laboratory of Aerospace Thermophysics, Ministry of Industry and Information Technology, Harbin Institute of Technology, 92 West Dazhi Street, Harbin, 150001 China; 2 Aarhus University, Department of Environmental Science/Interdisciplinary Centre for Climate Change, Frederiksborgvej 399, Roskilde, Denmark; 3 School of Mechanical Engineering, Beijing Institute of Technology, 100081 Beijing, China; 4 School of Urban Construction, Wuhan University of Science and Technology, No. 2, West Huangjiahu Road, Hongshan District, Wuhan, 430065 China; 5 Laboratory of Atmospheric Processes and Their Impacts, Ecole Polytechnique Fédérale de Lausanne (EPFL), Lausanne, Switzerland; 6 Center for the Study of Air Quality and Climate Change, Foundation for Research and Technology Hellas (FORTH), Thessaloniki, Greece

**Keywords:** Environmental impact, Environmental impact

## Abstract

This study estimated PM_2.5_ (atmospheric fine particulate matter with aerodynamic diameter ≤2.5 µg) concentrations and the health burden in mainland China from 2010 to 2049 under two scenarios: Current Legistaions and Maximum Technical Feasible Reductions. We assess premature deaths from PM_2.5_ exposure, examining sources like coal combustion, biomass burning, industry, and tailpipe emission from on-road transport. Results show that central and eastern China account for 75% of PM_2.5_-related deaths, with biomass burning (40%) and industry (34%) as primary contributors. Under the Current Legistaions and Maximum Technical Feasible Reductions scenarios, PM_2.5_-related premature deaths are projected to decrease by 43% and 80% (linear EVA) and by 28% increase and 40% decrease (nonlinear EVA) from 2010 to 2049. Assuming a linear relationship, the Maximum Technical Feasible Reductions scenario estimates that reduced PM_2.5_ exposure could avoid 1.55 million premature deaths annually by 2049 compared to 2010, primarily from coal combustion for heating, biomass burning, industry, and tailpipe emission from on-road transport.

## Main

With the rapid development of urbanization and industrialization, PM_2.5_ (atmospheric fine particulate matter with aerodynamic diameter ≤2.5 µg) pollution remains one of the major environmental problems facing China. As reported in the 2022 China Ecological and Environmental Conditions Bulletin^1^, 126 cities in China have exceeded ambient air quality standards (HJ 663-2013; Annual average PM_2.5_ concentration limit of 35 µg m^−3^, Daily average PM_2.5_ concentration limit of 75 µg m^−3^)^2^. Among them, the number of days with PM_2.5_ exceeding the standard is particularly serious, accounting for 36.9% of the total number of days with exceeding the standard. Meanwhile, PM_2.5_ poses a serious threat to human health^3^. According to the new state of global air report for 2024, in 2021, it has been estimated that 8.1 million people worldwide died due to indoor and outdoor air pollution^4,5^. The majority of these premature deaths occur in urban areas, where more than 50% of the global population—over 3.5 billion people—currently resides. According to the World Urbanization Prospects^6^, this figure is expected to rise to 70% by 2050 urbanization continues^7^. China, as one of the countries with a large population and high levels of air pollution, faces Dietary risk factors, high blood pressure, tobacco exposure, and ambient particulate matter pollution as the leading risk factor for premature death among its residents^8^. Previous research^9^ reported that in 2013, stroke, ischemic heart disease (IHD), lung cancer (LC), and chronic obstructive pulmonary disease (COPD) caused by air pollution accounted for 50%, 28%, 9%, and 12% of the total premature mortality in China, respectively. In response, China has implemented a series of air pollution control measures, such as the “Action Plan for the Battle Against Heavy Pollution Weather, Ozone Pollution Prevention and Control, and Diesel Truck Pollution Treatment”^10^, and the “Carbon Peak and Carbon Neutrality Goals”^11^, which have significantly reduced PM_2.5_ concentrations. However, according to the Global Burden of Disease 2019 comparative risk assessment, ambient PM_2.5_ pollution still led to approximately 1.4 million premature deaths in China in 2019^12^. Luo et al. similarly reported that shipping-related PM_2.5_ declined in all Chinese port cities under controls on sulfur emissions from shipping. However, the nationwide mortality rate from shipping-related, long-term exposure to PM_2.5_ increased by 11.4%, reaching 48,300 deaths by 2020^13^. To address this issue, China has made further efforts to improve air quality, and significant changes in future PM_2.5_ pollution levels and the associated health burden are anticipated. There is an urgent need for a quantitative assessment of the current and future health burden attributable to PM_2.5_ in China, which will be critical in informing the design of future air quality policies.

In health impact assessment models, the relationship between PM_2.5_ pollution and mortality is represented by exposure-response functions (ERFs)^14^. Traditionally, this relationship has been considered linear^15–17^. However, recent studies^18,19^ suggest that the ERFs for PM_2.5_ exposure and mortality may be nonlinear, with a steeper increase in the impacts at lower concentrations than at higher concentrations. Comparing the health burdens attributable to PM_2.5_ based on linear versus nonlinear ERFs provides potential insights into effectively avoiding premature deaths due to PM_2.5_ exposure. It also offers opportunities for enhancing regulatory capabilities, which may be a prerequisite for implementing more stringent measures.

Furthermore, mitigating the health impacts of PM_2.5_ pollution requires identifying the relative importance of various emission sources. Existing studies^20–24^ indicate that anthropogenic emission sources, including coal combustion, biomass burning, vehicular emissions, and industrial sources, are the predominant source categories across most regions of China under both historical and future business-as-usual scenarios. Anenberg et al. estimated the contribution of global transportation tailpipe emissions to the health burden associated with ambient PM_2.5_ in 2010 and 2015 using the GEOS-Chem model and epidemiological health impact assessment methods and reported that transportation emissions accounted for approximately 11.7% of global PM_2.5_- and ozone- related deaths in 2010 and 11.4% in 2015^25^. Reddington et al. explored the health benefits of eliminating emissions from different anthropogenic sources in South and East Asia in 2014 using the WRF-Chem model. They found that removing residential or industrial emissions could avoid 0.47 (95% confidence interval [CI]: 0.41–0.54) or 0.28 (95% CI: 0.236–0.36) million premature deaths annually, respectively, in India, China, and Southeast Asia^26^. Conibear et al. estimated the impact of future emission scenarios on air pollution exposure in China using the emulators, which were machine learning models trained and tested on simulator data from WRF-Chem. They found that, compared to the Current Legislation Emission (CLE) scenario in 2050, the application of the best air pollution technologies provides substantial health benefits, reducing the PM_2.5_-related health burden by 16%, avoiding 0.54 (95% CI: 0.5–0.57) million premature deaths per year. These public health benefits are mainly due to reductions in industrial (43%) and residential (30%) emissions^27^. Based on the WRF-Chem model and the Global Exposure Mortality Model (GEMM), Shen et al. assessed the impact of rural Chinese residential emissions on PM_2.5_ and health. They found that transitioning to clean energy significantly reduced contributions to ambient PM_2.5_, avoiding 0.13 (0.09–0.16) million premature deaths due to PM_2.5_ exposure. The climate forcing associated with this sector declines from 0.057 ± 0.016 W/m^2^ in 1992 to 0.031 ± 0.008 W/m^2^ in 2012^28^. Guo et al. evaluated the health benefits of air quality improvements following the deployment of clean energy in Hebei Province, China. They found that the pollutant emissions for 2030 could be reduced by 18–45% and 33–66% under the General Policy and Strengthened Policy scenarios, with the residential sector contributing the most. The General Policy scenario presented the moderate target of clean energy substitution, taking into account the economic feasibility and applicability of options and measures, while the Strengthened Policy scenario showed the ambitious target of clean energy deployment, which means the maximum potential to use clean energy in the energy-consuming sectors. Correspondingly, the health burden related to air pollution in 2030 would be reduced by 7499 and 12,260 cases, respectively, under these scenarios^29^.

In summary, the aforementioned studies demonstrate the significant benefits of controlling emissions from PM_2.5_ sources on air quality improvement and human health. However, previous research has mostly focused on the environmental health benefits of historical or future emission scenarios or has concentrated on specific regions within China. Few studies have considered (1) the contributions of various emission sources to the health burden in China over a long-term period from the past to future periods, and (2) the impacts of PM_2.5_ exposure-induced health benefits under nonlinear and linear relationships. Therefore, such studies are urgently needed to understand the extent to which different future scenarios will lead to improvements in air quality and health in China relative to the historical period, the extent to which specific emitting sources will contribute, and how the use of health impact assessment models for linear and nonlinear relationships affects the estimation of health burdens.

To address these issues, using the Danish Eulerian Hemispheric Model (DEHM) and the Economic Valuation of Air pollution (EVA) model, this study (1) estimated the number of premature deaths associated with PM_2.5_ pollution in mainland China during the historical period (2010–2014) and future periods (2020–2049) under CESM_SSP2-4.5_CLE (based on CESM_SSP2–4.5 meteorological data and Current Legislation Emission Scenario (CLE) emission assumptions) and CESM_SSP1-2.6_MFR (based on CESM_SSP1–2.6 meteorological data and Maximum Feasible Reductions (MFR) emission assumptions) scenario assumptions; (2) compared the impact of linear relationships (World Health Organization recommends a relative risk of 8.0%, referred to as WHO_RR) and nonlinear relationships on the health burden, explored the spatial distribution characteristics of PM_2.5_-related premature deaths, and quantified the impact of specific anthropogenic emission sources (coal combustion, biomass burning, industrial and transportation sources) on PM_2.5_-related premature deaths, thereby identifying their primary sources. This comprehensive study provides detailed source information necessary for prioritizing PM_2.5_ air pollution control and reducing health risks associated with PM_2.5_.

## Results

### Future PM_2.5_ concentrations and population projections

**Fig. 1:**
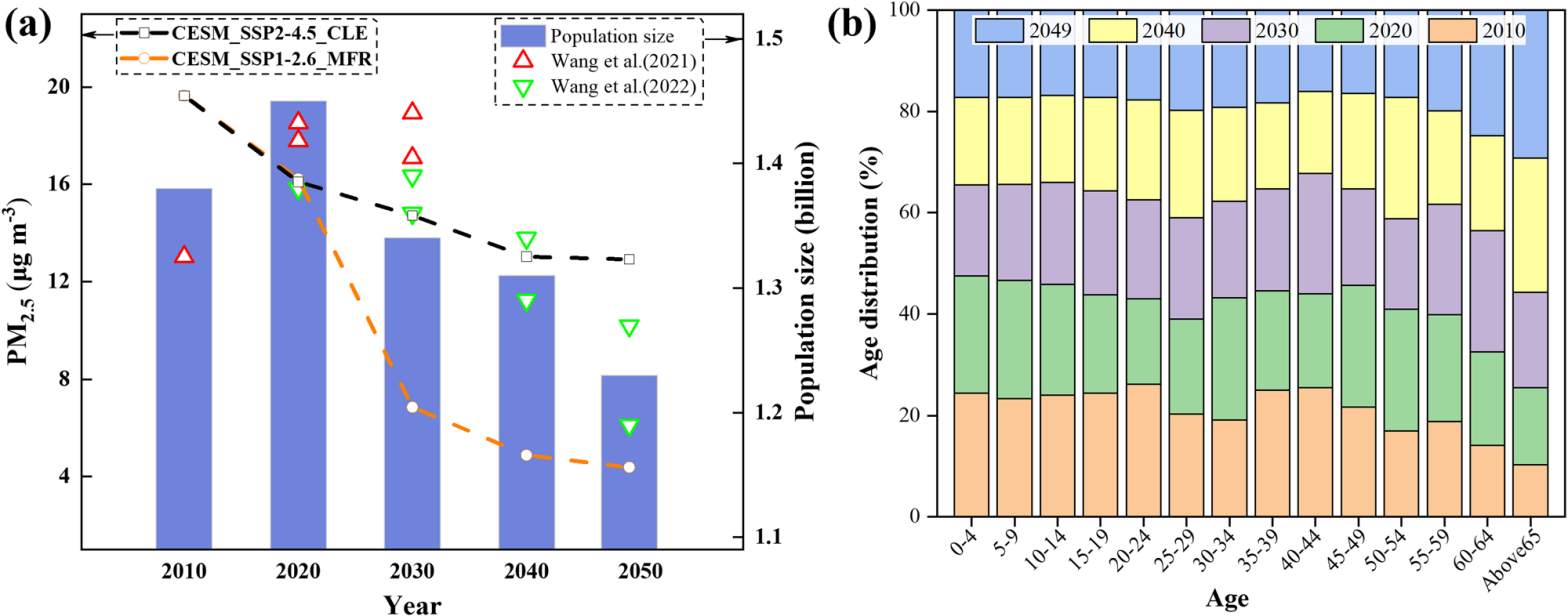
Projection of annual average PM_2.5_ concentration population sizes and age distributions in China from 2010 to 2049. Subplots show the projection of annual average PM_2.5_ concentration and population sizes (**a**), and age distributions (**b**) in China from 2010 to 2049. Purple bars and red and green triangles (**a**) represent population size; black and orange line graphs (**a**) represent PM_2.5_ concentrations under CESM_SSP2-4.5_CLE and CESM_SSP1-2.6_MFR scenarios, respectively. Blue, yellow, purple, green, and orange rectangles (**b**) represent the age distributions in 2049, 2040, 2030, 2020, and 2010, respectively.

Figure 1a shows the projected changes in the total population size of China from 2010 to 2049 and the changes in the annual mean PM_2.5_ concentration changes under the CESM_SSP2-4.5_CLE scenario and CESM_SSP1-2.6_MFR scenario. Figure 1b shows the age distributions in China from 2010 to 2049. The predicted PM_2.5_ concentrations and population sizes will serve as the basis for subsequent research to better assess China’s environmental health impacts. From Fig. 1a, it can be found that the total population size is expected to peak at 1.45 billion in 2020, and the trend of the predicted results in this work is comparable to those of Wang et al.^30^ and Wang et al.^31^, with a maximum difference of 7%. Figure 1b illustrates the projected increase in the proportion of the population aged 65+ from 8% in 2010 to 23% in 2049, an increase of about 170 million. Compared to 2020, the population of persons aged 65 years or older in 2049 will increase by about 110 million. Additionally, this study forecasts that PM_2.5_ concentrations under both scenarios will show a declining trend to varying degrees starting from 2010. Under the CESM_SSP2-4.5_CLE scenario, PM_2.5_ concentrations are expected to decrease by 6.7 μg m^−3^, approximately 34%, from 2010 to 2049. In contrast, the CESM_SSP1-2.6_MFR scenario predicts a more significant reduction in PM_2.5_ concentrations by 15.3 μg m^−3^, approximately 78% (see details in Liu et al.^32^).

### Health burden analysis

**Fig. 2:**
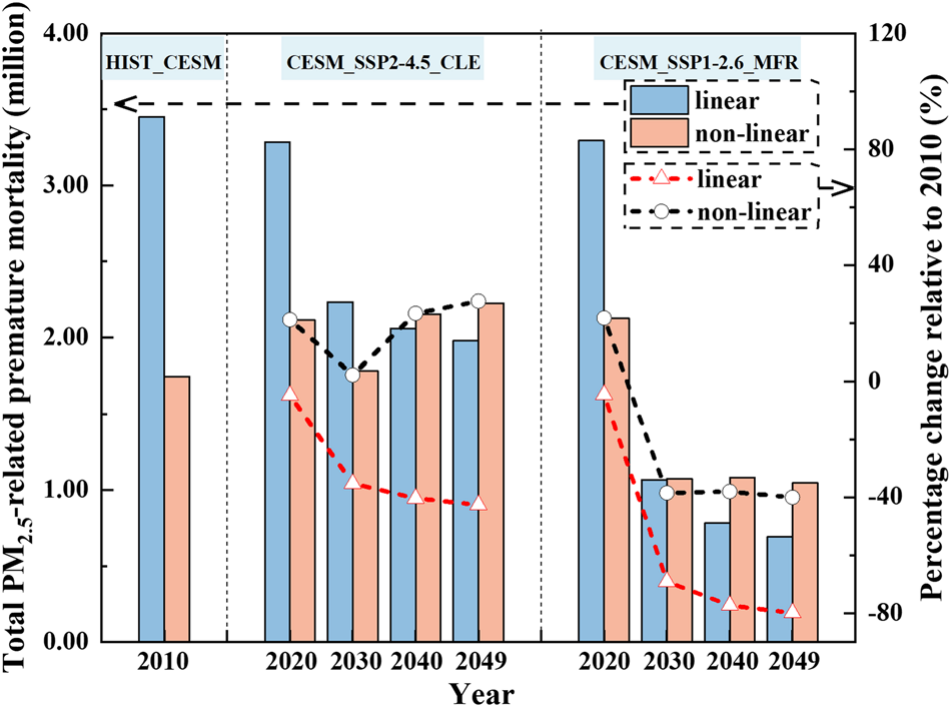
Total PM_2.5_-related premature mortality and its percentage change relative to 2010 in mainland China estimated with the linear EVA (RR = 8.0%, recorded as WHO_RR) and nonlinear EVA from 2010 to 2049 under different scenarios. Three zones from left to right represent HIST_CESM, CESM_SSP2-4.5_CLE, and CESM_SSP1-2.6_MFR scenarios. Blue and orange bars represent the total PM_2.5_-related premature mortality estimated by linear and nonlinear EVA. Red and black line plots represent the percent change estimated by linear and nonlinear EVA.

Acute deaths, chronic deaths, and total deaths attributable to PM_2.5_ pollution were estimated based on the linear EVA (WHO_RR), and PM_2.5_-related cause-specific deaths were estimated using the nonlinear EVA, including lung cancer, chronic obstructive pulmonary disease, ischemic heart disease, stroke, lower respiratory infections, and and the sum of these as the total deaths. Detailed PM_2.5_ pollutant-related causes of death in mainland China under different scenarios are summarized in Table 1. Figure 2 depicts the estimates of premature deaths related to PM_2.5_ in mainland China from 2010 to 2049 under different scenarios based on linear EVA (WHO_RR) and nonlinear EVA, along with their percentage changes relative to 2010.

**Table 1:** Premature deaths attributable to PM_2.5_ pollutants in mainland China under different scenarios (in million)

Model	Premature deaths (million)	HIST	CESM_SSP2-4.5_CLE				CESM_SSP1-2.6_MFR			
		_CESM								
		2010	2020	2030	2040	2049	2020	2030	2040	2049
Linear EVA	AD	0.37	0.32	0.21	0.19	0.18	0.32	0.10	0.07	0.06
	CD	3.08	2.96	2.02	1.87	1.80	2.97	0.97	0.71	0.63
	Total deaths	3.45	3.28	2.23	2.06	1.98	3.29	1.07	0.78	0.69
Non-linear EVA	LC	0.17	0.22	0.19	0.22	0.23	0.23	0.10	0.09	0.09
	COPD	0.29	0.29	0.26	0.33	0.34	0.29	0.14	0.14	0.14
	IHD	0.47	0.60	0.54	0.65	0.67	0.60	0.34	0.34	0.33
	stroke	0.15	0.18	0.15	0.19	0.19	0.18	0.07	0.07	0.06
	LRI	0.07	0.09	0.08	0.10	0.11	0.09	0.04	0.04	0.04
	Total deaths	1.75	2.11	1.78	2.15	2.23	2.12	1.07	1.08	1.05

*AD* corresponds to acute deaths due to short-term exposures, *CD* to chronic deaths due to long-term exposures, *LC* to lung cancer, *COPD* to chronic obstructive pulmonary disease, *IHD* to ischemic heart disease, and *LRI* corresponds to lower respiratory infections.

PM_2.5_ pollution-related causes of death, including AD (acute deaths), CD (chronic deaths), and ADCD (total premature deaths; ADCD = AD + CD), estimated from linear EVA, show a decreasing trend under both scenarios. The projections indicate that compared with 2010, the reduction of AD, CD, and total deaths cases estimated based on the WHO_RR in 2049 amounted to 52%, 42%, and 43%, respectively under the CESM_SSP2-4.5_CLE scenario; the reduction of AD, CD, and total deaths cases estimated based on the WHO_RR in 2049 amounted to 83%, 80%, and 80%, respectively under the CESM_SSP1-2.6_MFR scenario. It is worth noting that compared with 2010, the total population of China in 2030, 2040 and 2049 will decrease by 2%, 5% and 11% respectively (Fig. 1). This reveals that decreases in the number of deaths associated with PM_2.5_ pollution outpaces changes in the population. In addition, the results indicate that the reductions in AD, CD, and total deaths are more significant under the CESM_SSP1-2.6_MFR scenario compared to the CESM_SSP2-4.5_CLE scenario, due to stricter PM_2.5_ emission controls. For individual causes of death, AD shows a significant reduction under all scenarios, suggesting that emergency measures to prevent PM_2.5_ pollution will yield substantial health benefits.

The PM_2.5_-related causes of death estimated based on non-linear EVA, including LC, COPD, IHD, stroke, LRI, and total deaths, all showed a small upward trend in the CESM_SSP2-4.5_CLE scenario (Table 1 and Fig. 2). Due to more aggressive pollution emission controls, the numbers of deaths estimated by nonlinear EVA show a decreasing trend under the CESM_SSP1-2.6_MFR scenario (Table 1 and Fig. 2). The main reason for the different trends in causes of death under the two scenarios may be related to the combination of different PM_2.5_ concentration levels and distributions and development in population age distribution. As shown in Fig. 1a, compared with the CESM_SSP2-4.5_CLE scenario, the PM_2.5_ concentration decreases more dramatically in the CESM_SSP1-2.6_MFR scenario, and the corresponding PM_2.5_-related causes of death is more effectively controlled. In addition, as shown in Fig. 1b, the proportion of the population aged 65+ is projected to increase from 8% in 2010 to 23% in 2049, an increase of about 170 million. Therefore, in the CESM_SSP2-4.5_CLE scenario, there is a possibility that air quality improvements in some cities have not yet reached the point of fully offsetting the health risks associated with the increase in the elderly population^33^, leading to a slow-growth trend in premature death cases. In the CESM_SSP1-2.6_MFR scenario, on the other hand, improved air quality offsets the health risks associated with an increasing elderly population, contributing to a downward trend in premature deaths. The projections indicate that compared with 2010, the increases in LC, COPD, IHD, stroke, LRI, and total deaths cases estimated based on the nonlinear EVA in 2049 under the CESM_SSP2-4.5_CLE scenario are 39%, 21%, 43%, 32%, 48%, and 28%, respectively; the reduction in LC, COPD, IHD, stroke, LRI, and total deaths cases estimated based on the nonlinear EVA in 2049 under the CESM_SSP1-2.6_MFR scenario are 46%, 52%, 30%, 56%, 48%, and 40%, respectively, compared to 2010. For specific causes of death, the number of stroke cases estimated based on nonlinear EVA is the most sensitive to PM_2.5_ changes under the CESM_SSP1-2.6_MFR scenario, with the largest decrease.

The above causes of death estimated based on linear and nonlinear EVA show different trends in the CESM_SSP2-4.5_CLE scenario. This is mainly related to the different compositions of the nonlinear and linear functions themselves, together with different PM_2.5_ concentration levels and distributions and population age distributions, thus leading to different trends. The impact of the assumption of linear or nonlinear relationships on the estimated total premature deaths related to PM_2.5_ is explored based on Fig. 2 and Table 1. The results show that the average annual total PM_2.5_-related deaths estimated from the linear EVA are higher than those from the non-linear EVA in both 2010 and 2020. It may be related to the nonlinearity of the concentration-response function. Some studies^18,19^ have found a non-linear relationship between air pollution and mortality, with health effects increasing faster at lower concentrations than at higher concentrations. This also means that linear relationships can lead to overestimates of health effects in highly polluted areas. Therefore, when applying linear EVA to China with high PM_2.5_ pollution in 2010 and 2020, it may lead to an increase in linear estimates of premature death.The annual average total deaths related to PM_2.5_ from 2020 to 2049, estimated based on linear and nonlinear EVA, are approximately 2.39 million and 2.07 million cases under the CESM_SSP2-4.5_CLE scenario; the annual average total deaths related to PM_2.5_ are approximately 1.46 million and 1.33 million cases under the CESM_SSP1-2.6_MFR scenario. In addition, it was found that compared to the linear EVA, the annual average change in PM_2.5_-related premature deaths from 2020 to 2049 under the CESM_SSP2-4.5_CLE scenario, estimated based on the nonlinear EVA is projected to be −13%. compared to the linear EVA; and the annual average change under the CESM_SSP1-2.6_MFR scenario is projected to be −9%.

To date, many studies have estimated PM_2.5_-related deaths in China. For instance, Li et al. used the IER model to estimate that the number of deaths related to PM_2.5_ in mainland China in 2008 was 1.14 million^33^. Similarly, Wu et al. used the GEMM model to estimate that PM_2.5_-related deaths in China in 2015 were 1.92 million^34^. This result is comparable to the total deaths related to PM_2.5_ estimated by nonlinear EVA in this study, approximately 1.8 million cases in 2010 and 2.1 million cases in 2020, but lower than the estimates based on linear EVA. The total number of premature deaths related to PM_2.5_ in 2010 estimated by linear EVA was approximately 3.5 million as shown in Fig. 2. The absolute values of the percentage change in total premature deaths estimated by EVA relative to the CESM_SSP2-4.5_CLE scenario increase year by year under the CESM_SSP1-2.6_MFR scenario (Supplementary Table 1). This result is also similar to the percentage change in PM_2.5_ (Supplementary Table 1), indicating that controlling PM_2.5_ emissions helps alleviate PM_2.5_-related diseases.

Regarding the primary causes of death, the results of premature death cases estimated based on the linear EVA model, show (Table 1) that chronic deaths is the leading cause of death under both scenarios, accounting for approximately 90% of total deaths during the study period. The results estimated based on nonlinear EVA (Table 1) show that ischemic heart disease is the primary cause of death under both scenarios, accounting for approximately 41–50% of the combined total deaths from the five causes (lung cancer, chronic obstructive pulmonary disease, ischemic heart disease, stroke, lower respiratory infections) during the study period. Similar results have been reported by Liu et al.^35^.

### Spatial distribution of total PM_2.5_-related premature mortality

**Fig. 3:**
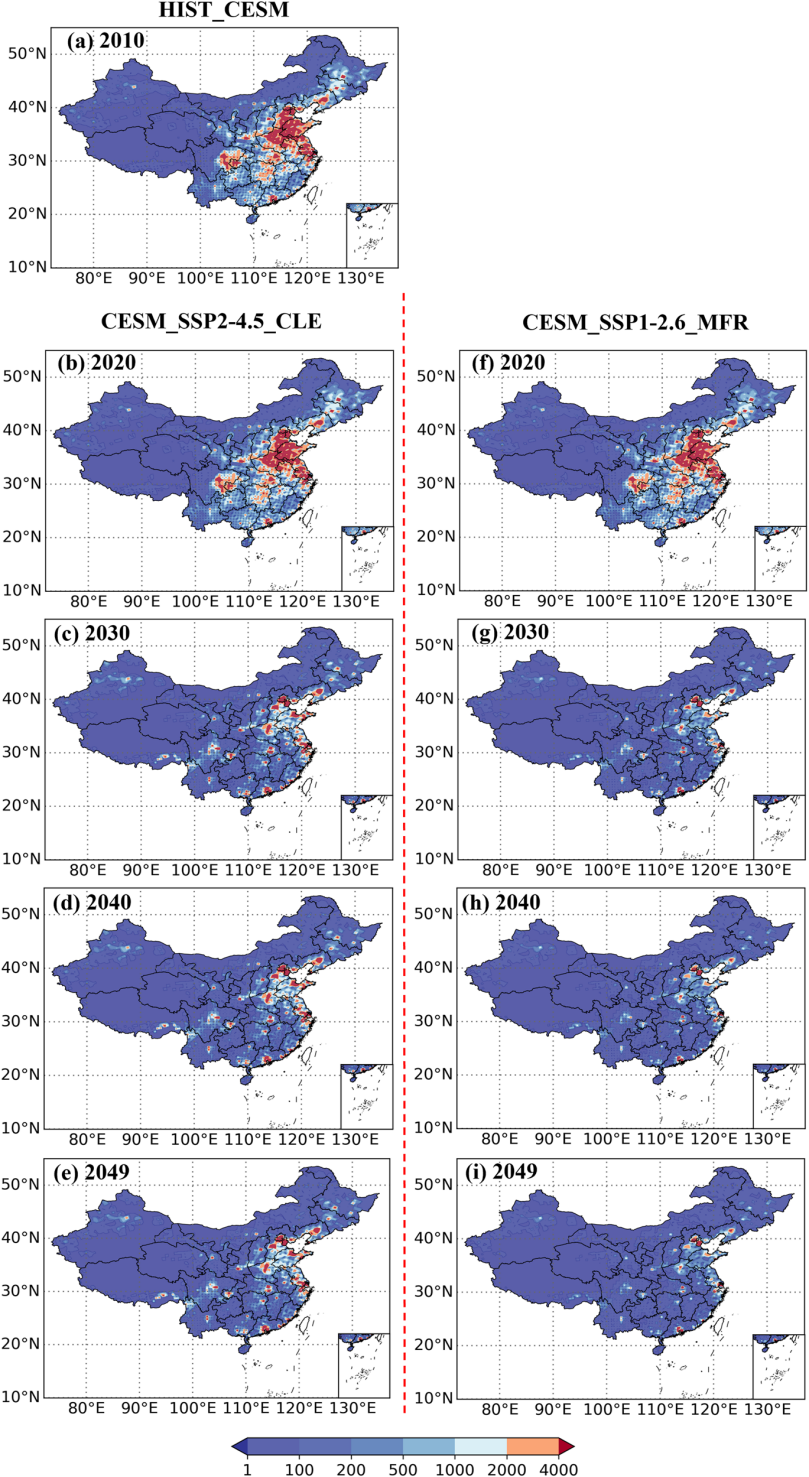
Spatial distribution of PM_2.5_-related premature mortality estimated with the linear EVA (RR = 8.0%, recorded as WHO_RR) in mainland China from 2010 to 2049 under different scenarios (in case). The color scale is logarithmic. **a** refer to the HIST_CESM scenario in 2010, **b**–**e** represent the CESM_SSP2-4.5_CLE scenario during the period from 2020 to 2049, while (**f**–**i**) denote the CESM_SSP1-2.6_MFR scenario in the same period.

**Fig. 4:**
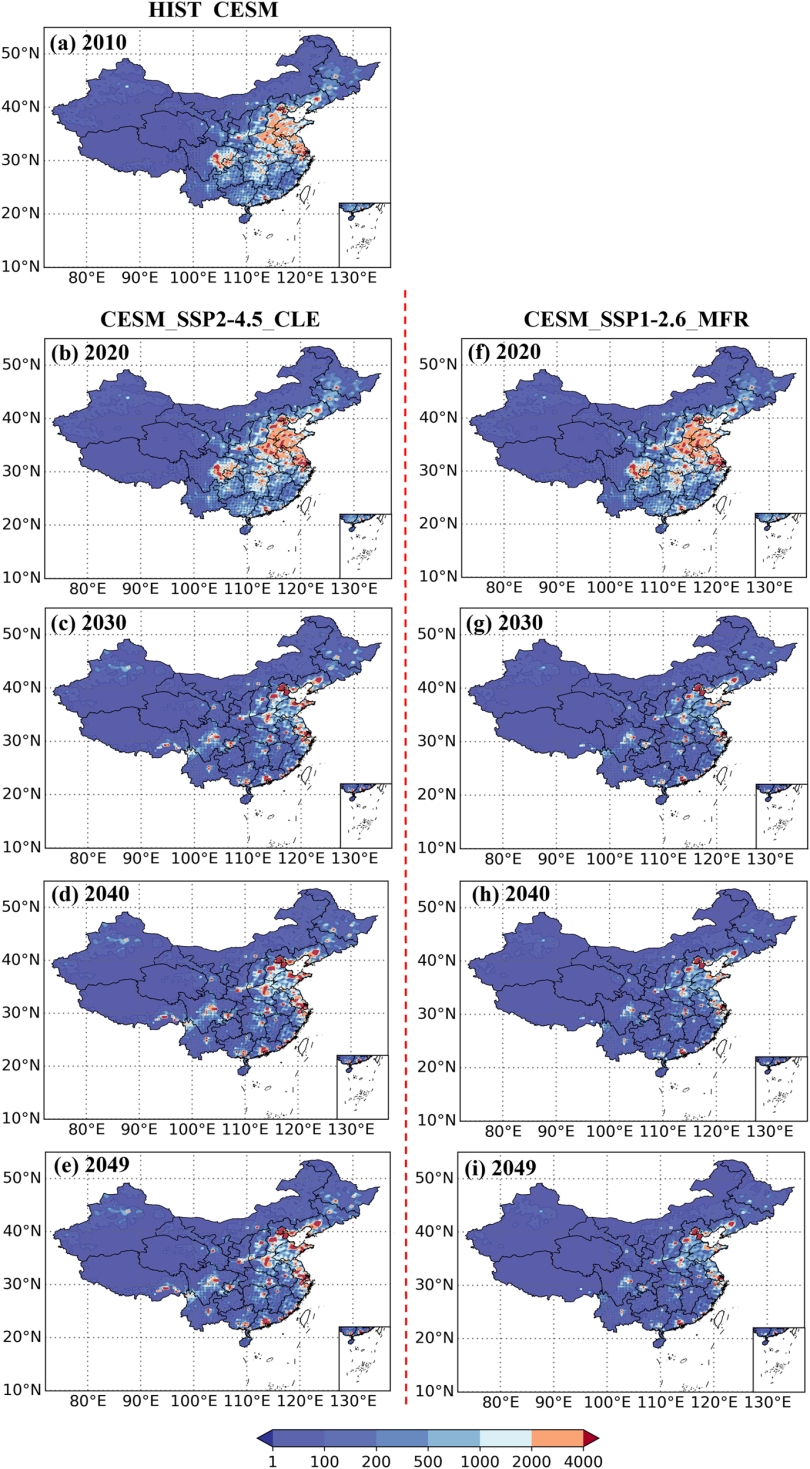
Spatial distribution of PM_2.5_-related premature mortality estimated with the nonlinear EVA in mainland China from 2010 to 2049 under different scenarios (in case). The color scale is logarithmic. The color scale is logarithmic. **a** refer to the HIST_CESM scenario in 2010, **b**–**e** represent the CESM_SSP2-4.5_CLE scenario during the period from 2020 to 2049, while **f**–**i** denote the CESM_SSP1-2.6_MFR scenario in the same period.

Figures 3 and 4 show the spatial distribution of premature deaths related to PM_2.5_ in mainland China from 2010 to 2049, estimated using the linear EVA (WHO_RR) and nonlinear EVA under the different scenarios. Figures (a) refer to the HIST_CESM scenario in 2010, figures (b~e) represent the CESM_SSP2-4.5_CLE scenario during the period from 2020 to 2049, while figures (f~i) denote the CESM_SSP1-2.6_MFR scenario in the same period. Overall, the high values of PM_2.5_-related premature deaths are concentrated in central and eastern China (see Supplementary Fig. 1 for a geographic location), which accounted for about 75% of the total PM_2.5_-related premature deaths in China (Supplementary Table 2). Except for the premature deaths estimated by the nonlinear EVA under the CESM_SSP2-4.5_CLE scenario, the implementation of the CESM_SSP2-4.5_CLE and CESM_SSP1-2.6_MFR scenarios can effectively reduce PM_2.5_-related premature deaths from 2010 to 2049. Calculations show that under the CESM_SSP2-4.5_CLE and CESM_SSP1-2.6_MFR scenarios, PM_2.5_-related premature deaths are expected to decrease by up to 43% and 80% by 2049 compared to 2010.

### Source contributions to total PM_2.5_-related premature mortality

**Fig. 5:**
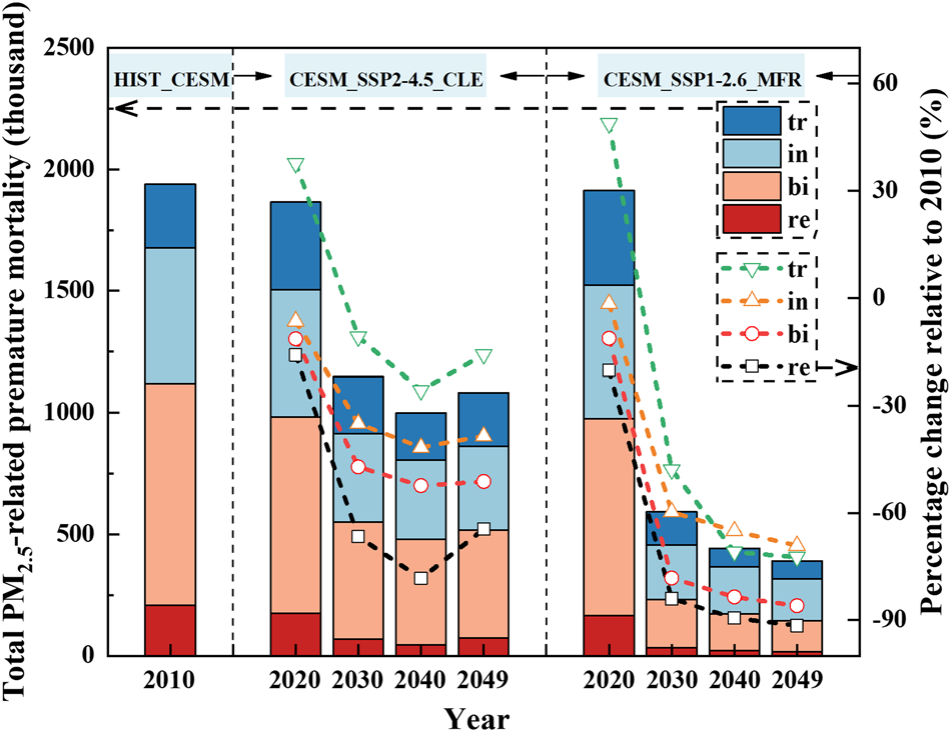
Absolute contributions of anthropogenic emission sources to total PM_2.5_-related premature mortality estimated with the linear EVA (RR = 8.0%, recorded as WHO_RR) and their percentage change relative to 2010 in mainland China under different scenarios from 2010 to 2049. Anthropogenic emission sources include coal combustion for residential heating (re), biomass burning (bi), industry (in), and tailpipe emission from on-road transport (tr). Three zones from left to right represent HIST_CESM, CESM_SSP2-4.5_CLE, and CESM_SSP1-2.6_MFR scenarios. Dark blue, light blue, orange, and red rectangles represent the absolute contributions of tailpipe emission from on-road transport (tr), industry (in), biomass burning (bi), and coal combustion for residential heating (re) to total PM_2.5_-related premature deaths, respectively. Green, orange, red, and black line plots represent the contribution of tailpipe emission from on-road transport, industry, biomass burning, and coal combustion for residential heating to the percentage change, respectively.

Based on the new relative risk value WHO_RR recommended by WHO, the impacts of specific emission sources on premature deaths related to PM_2.5_ were investigated using the linear EVA model. Figure 5 depicts the estimated premature deaths attributable to PM_2.5_ from various emission sources in mainland China from 2010 to 2049 under the HIST_CESM, CESM_SSP1-2.6_MFR, and CESM_SSP2-4.5_CLE scenarios, based on linear EVA (WHO_RR). The left Y-axis represents the premature deaths caused by PM_2.5_ from each emission source, while the right Y-axis represents the percentage change relative to 2010. Under the CESM_SSP2-4.5_CLE scenario, the percentage change in premature deaths caused by PM_2.5_ from the coal combustion for residential heating (re), biomass burning (bi), industrial emission (in), and tailpipe emission from on-road transport (tr) in mainland China in 2049 estimated with the linear EVA is projected to be –65%, –51%, –38%, and –16%, respectively, relative to 2010.Under the CESM_SSP1-2.6_MFR scenario, the percentage change in premature deaths caused by PM_2.5_ from the coal combustion for residential heating, biomass burning, industrial emission, and tailpipe emission from on-road transport in mainland China in 2049 estimated with the linear EVA is projected to be –92%, –86%, –69%, and –72%, respectively, relative to 2010. It is estimated that compared to the CESM_SSP2-4.5_CLE scenario, the implementation of more stringent scenarios from 2030 to 2049 will contribute to reducing premature deaths caused by each emission source by 38–76%. These results indicate that compared to the CESM_SSP2-4.5_CLE scenario, the CESM_SSP1-2.6_MFR scenario is expected to effectively avoid premature deaths attributable to PM_2.5_ from various emission sources in the future.

**Fig. 6:**
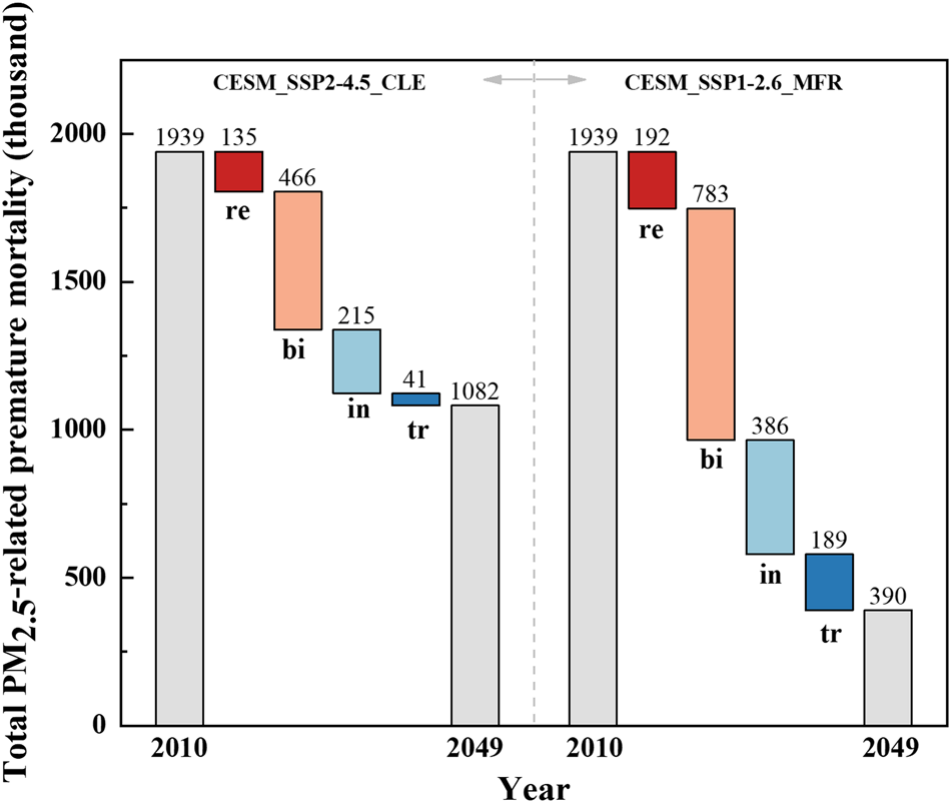
The inter-annual variations of anthropogenic-driven total PM_2.5_-related premature mortality estimated with the linear EVA (RR = 8.0%, recorded as WHO_RR) under different scenarios in mainland China. Dark blue, light blue, orange, and red rectangles represent the inter-annual variations of total PM_2.5_-related premature mortality driven by tailpipe emission from on-road transport (tr), industry (in), biomass burning (bi), and coal combustion for residential heating (re), respectively. Two zones from left to right represent CESM_SSP2-4.5_CLE, and CESM_SSP1-2.6_MFR scenarios. PM_2.5_-related premature deaths in 2010 and 2049 refer to the sum of PM_2.5_-induced premature deaths from re, bi, in, and tr sources in the corresponding years.

Supplementary Fig. 2 shows the relative contribution of various emission sources in mainland China to the PM_2.5_-related premature deaths estimated using linear EVA for the period 2010–2049 under different scenarios. Regarding the average annual contribution levels of emission sources for the period 2020~2049, the results indicate that the biomass burning (40%) and industrial (34%) emission sources are the main contributors to PM_2.5_-related premature deaths estimated using linear EVA under all scenarios. Therefore, the health benefits or risks associated with reductions in PM_2.5_ exposure under the scenarios assuming implementation of PM_2.5_ emission controls are primarily from biomass burning and industrial emissions. Moreover, the changes in emission source-driven PM_2.5_-related premature deaths estimated using the linear EVA (WHO_RR) model for the period 2010~2049 are shown in Fig. 6. The PM_2.5_-related premature deaths in 2010 and 2049 refer to the sum of PM_2.5_-induced premature deaths from the coal combustion for residential heating, biomass burning, industrial emission, and tailpipe emission from on-road transport in the corresponding years. The results indicate that, under all scenarios, the primary contributor to the reduction in PM_2.5_-related premature deaths from 2010 to 2049 is the biomass burning source. Specifically, the predicted results are as follows: under the CESM_SSP2-4.5_CLE and CESM_SSP1-2.6_MFR scenarios, PM_2.5_-related premature deaths from the coal combustion for residential heating, biomass burning, industrial emission, and tailpipe emission from on-road transport are expected to decrease by 0.86 million and 1.55 million cases from 2010 to 2049, with 54% and 51% of these reductions attributable to the control of biomass burning source emissions in the corresponding scenarios. Furthermore, as indicated by the results in Fig. 2, the total PM_2.5_-related premature deaths under the CESM_SSP2-4.5_CLE and CESM_SSP1-2.6_MFR scenarios are projected to decrease by 1.47 million and 2.76 million cases from 2010 to 2049. This demonstrates that the reduction in overall PM_2.5_-related premature deaths during the study period is not solely due to the emission control of the four sources mentioned above. These findings suggest that, in addition to focusing on PM_2.5_ emissions from the coal combustion for residential heating, biomass burning, industrial emission, and tailpipe emission from on-road transport, future research should also consider other sources of emissions not covered in this work, such as other anthropogenic and natural emissions.

## Discussion

We propose to synthesize the linear or nonlinear relationship between PM_2.5_ concentrations and mortality and give a more comprehensive projection of future premature deaths. Based on the DEHM model, we designed sensitivity experiments to capture PM_2.5_ pollution in various Chinese cities under different emission reduction scenarios in the future. By assuming linearity and nonlinearity between PM_2.5_ concentration and mortality in the EVA model, we found that the linear and nonlinear relationship between the two has less impact on the results of health impact assessment. The annual mean change in total PM_2.5_-related mortality estimated from the linear and nonlinear relationships ranged from 9% to 13% under both scenarios. This is also largely attributable to China’s aging population. Our projections indicate that the proportion of the population over 65 years of age is projected to increase from 8% in 2010 to 23% in 2049, an increase of about 170 million. This proportion plays an important role in the linear and nonlinear relationship. It is worth noting that population aging is also an important challenge for China to deal with air pollution in the future^36^. More attention should be paid to aging in the future.

The results of the health impact assessment indicate that strict pollution emission controls on anthropogenic sources are an effective way to significantly alleviate the PM_2.5_-related mortality burden. Compared to the CESM_SSP2-4.5_CLE scenario, implementing more stringent hypothetical scenarios during 2030–2049 will help reduce premature deaths from PM_2.5_ emissions across various sources by 38–76%. Using linear EVA estimates, the implementation of the CESM_SSP2-4.5_CLE and CESM_SSP1-2.6_MFR scenarios from 2010 to 2049 would avoid 0.86 million and 1.55 million PM_2.5_-related premature deaths from the coal combustion for residential heating, biomass burning, industry, and tailpipe emission from on-road transport, with approximately 54% and 51% achieved through controlling biomass burning source emissions in the corresponding scenarios. Therefore, there is an urgent need for China to implement further measures to effectively reduce biomass burning emissions. The relevant study^37^ has proposed that the use of advanced stoves, such as gasified biomass cooking stoves for biomass and “under-fire” heating stoves for a variety of solid fuels, can reduce pollutant emissions from domestic coal and biomass combustion by improving combustion and thermal efficiency. Improving solid fuel quality, such as including fuel volatile matter content, fuel size and fuel form, is also one of the possible ways to reduce pollutant emissions from domestic coal and biomass burning^37^. In addition, China should, on the one hand, continue or intensify the comprehensive utilization of straw and the ban on burning (from the Action Plan for Sustained Improvement of Air Quality^38^) to reduce biomass burning emissions and the health burden caused by air pollution. On the other hand, potential emerging technologies, including energy storage technologies, should be developed. For example, the deployment of heat storage devices (e.g., large-scale water heat storage tanks) in centralized biomass heating systems is encouraged to balance heating demand and fuel supply to avoid high pollution from peak combustion; and the efficiency of biomass gasification to hydrogen or liquefied fuels can be enhanced through energy storage technologies to reduce carbon emissions from traditional fuel production processes.

This study benefited from having data on PM_2.5_ concentrations from various anthropogenic emission sources spanning 30 years and based on EVA model to synergistically consider the linear or non-linear relationship between PM_2.5_ concentrations and mortality. The aim is to fully reveal the health benefits of future emission reduction measures. However, uncertainties may arise due to uncertainties in the exposure-response-functions, the incomplete consideration of species in the health impact assessment and the incomplete inclusion of emission sources and uncertainties in emissions in the DEHM model applied to China.

Regarding the health impact assessment, the current EVA system considers the following compounds related to human health impacts: O_3_, NO_2_, SO_2_, SO_4_^2−^, NO_3_^−^, NH_4_^+^, primary emissions of PM_2.5_. and secondary organic aerosols (SOA)^39^. For the secondary inorganic components, the full weight of the particles including nitrate, sulfate and ammonium is applied as follows: Besides SO_4_^2−^ the full weight of H_2_SO_4_, NH_4_SO_4_ and (NH_4_)_2_SO_4_ are included. The same applies for NO_3_^−^ where also NH_4_NO_3_ is included^39^. Primary particles include dust, elemental carbon (both fresh and aged), organic matter (OM) and sea salt^39^. Regarding baseline mortality data selection, using 2017 baseline mortality data for future scenario projections could affect future changes in premature mortality associated with PM_2.5_. Nonetheless, this study considered changes in population and PM_2.5_ concentrations under future scenarios, which reduces uncertainty in the results of future health impact assessments. It is important to note that, while the linear or nonlinear exposure-response relationships between PM_2.5_ concentrations and health burdens may introduce uncertainties, the strength of this study lies in its simultaneous evaluation of health burdens estimated based on both linear and nonlinear relationships. This comprehensive approach allows for a thorough assessment of the maximum anticipated health benefits from the implementation of the hypothetical scenarios in mainland China. Specifically, for the cases discussed in this paper, the upper limit of health benefits for the period 2010–2049 is estimated to potentially avoid approximately 2.76 million PM_2.5_-related premature deaths in mainland China (Fig. 2), annually. Regarding the predicted PM_2.5_ concentrations, a rather low resolution could add to the uncertainty of the exposure. Morover, the DEHM model does not include all PM_2.5_ emission sources, such as wind blown dust/sand emissions, which are predominantly concentrated in western China^40^. This omission could lead to an underestimation of predicted concentrations, however, since these are natural emissions, it will not influence the scenarios. Since the major PM_2.5_ pollution and population in mainland China are mainly concentrated in the central-eastern region of China, the number of premature deaths is also in these regions, consistent with the findings of this study and Li et al.^41^. Given the focus of this study on the impacts of anthropogenic emission sources (coal combustion for residential heating, biomass burning, industrial emission, and tailpipe emission from on-road transport) on PM_2.5_-related premature deaths, the impact of dust emissions was not considered. To justify this omissions, previous study results^34,35^ were compared in “*Health burden analysis*”: the total PM_2.5_-related deaths estimated using the nonlinear EVA in this study are comparable to those of Wu et al.^34^, with a maximum difference of 11%, indicating that the impact of excluding dust emissions is within an acceptable range. In the DEHM model, the transportation source only considers tailpipe emissions from road transport, excluding non-tailpipe emissions from vehicles like road dust, brake dust, and tire wear, as well as other transportation sources like ships and airplanes. This is because tailpipe emissions from road transport have a more significant impact on air quality and its associated health risks than emissions from other transportation sources like ships and airplanes^42–45^. Similarly, comparisons with the existing literature were made to reveal the plausibility of this study’s assessment of the impact of tailpipe emission from on-road transport: Zheng et al.^46^ assessed the contribution of sources of PM_2.5_-related premature deaths in China and showed that the contribution of vehicle emissions to the premature deaths due to PM_2.5_ pollution increased from 8% in 2005 to 14% in 2015, which is similar to the results of this study. The contribution of tailpipe emission from on-road transport to PM_2.5_-related premature mortality ranges from 14% to 23% from 2010 to 2049 under the CESM_SSP2-4.5_CLE and CESM_SSP1-2.6_MFR scenarios in this study (Supplementary Fig. 2). Among them, the contribution of tailpipe emissions from on-road transport was 14% in 2010. Therefore, the contribution of tailpipe emissions from on-road transport to PM_2.5_-related premature deaths in this study can be considered reasonable. In conclusion, the methodology employed in this study is reliable for estimating current and future PM_2.5_-related premature deaths, assessing the health benefits of pollutant abatement measures, and identifying the specific contributions of various anthropogenic emission sources. This approach also provides a reference for future studies aiming to estimate environmental and health benefits under different PM_2.5_ control scenarios in different countries and regions. Future research will further consider a broader range of species and sources on health impacts, enhance the resolution of the DEHM model, and propose scenarios and criteria for determining the applicability of linear and nonlinear models to accurately predict future PM_2.5_-related premature deaths.

## Methods

### Meteorological data and emission inventories

Coupled Model Intercomparison Project Phase 6 (CMIP6) includes a historical period covering the years 1850–2014, and a future period from 2015 to 2100, providing an important data foundation for assessing models’ ability to simulate past and current climate change and for projecting future climate change^47,48^. Wang et al.^31^ also adopted the above rules, dividing 1950–2014 into the historical period and 2015–2100 into the future period. Accordingly, this study adheres to the CMIP6 division between historical and future periods, considering the historical years of 2010–2014, as well as future years of 2020 (near-term), 2030 (mid-term), 2040, and 2049 (long-term). From a temporal perspective, we investigate the trends of PM_2.5_ concentrations and its resulting health burden in China during the future period. The meteorological data used as input to the DEHM model, is obtained from the numerical weather prediction model WRFv4.1^49^ and driven by global reanalysis or global climate simulation. For 2010–2014, WRF is driven by global meteorological data obtained from the ERA5 dataset and the Community Earth System Model (CESM) global model, respectively. Notably, CESM is one of the models participating in CMIP6. In this study, based on SSP2-4.5 and SSP1-2.6 of the shared socioeconomic pathways (SSPs), we utilized the CESM to simulate and predict future climates in China from 2020 to 2049 under two scenarios: CESM_SSP2-4.5 and CESM_SSP1-2.6. Specifically, the SSP2-4.5 scenario represents a moderate radiative forcing scenario that continues the historical development pattern throughout the 21st century, with radiative forcing stabilizing at 4.5 W/m^2^ by 2100^31^. As for the SSP1-2.6 scenario, a relatively optimistic trend in human development, characterized by substantial investments in education and health, robust economic growth, and well-functioning organizational operations are assumed. This scenario is progressively transitioning towards sustainability-related measures, with radiative forcing stabilizing at 2.6 W/m^2^ by 2100^31^. In addition, there are some differences between the two scenarios described above in terms of urbanization projections and assumptions about economic decarbonization. For example, in the urbanization projections: the SSP1-2.6 scenario assumes rapid urbanization in all country groups associated with high income growth^50^. The SSP2-4.5 scenario assumes a moderate rate of urbanization growth around the world compared to the historical experience of similar countries^50^. In terms of scenarios for decarbonizing the economy: SSP1-2.6 scenario assumes that economic growth is decoupled from energy demand through improved energy efficiency and increased use of renewable energy^51^. The scenario features low social acceptability of other conventional technologies (such as fossil fuel conversion technologies, nuclear power, and carbon dioxide capture and storage) except non-biomass renewables, which also feature fast technological improvements^51^. In addition, the adoption of carbon dioxide capture and storage in the power sector will have an impact on future PM_2.5_ emissions from this sector. The SSP2-4.5 scenario generally assumes that all technologies develop and are socially acceptable at medium levels^51^. All CMIP6_CESM results can be obtained from the official CMIP6 archive (https://esgf-node.llnl.gov/search/cmip6/).

The emission inventory is obtained from Eclipse v6b^52^. The future emission projections in this dataset are based on explicit assumptions about economic development and the effectiveness of specific emission control policies. The Eclipse v6b dataset offers two emission reduction scenarios, both of which were utilized in this study: the Current Legislation Emission Scenario (CLE) and Maximum Feasible Reductions (MFR). The CLE scenario is applied throughout the study period, encompassing both historical and future periods. It is based on the assumption that future emission levels will continue to adhere to existing laws and policies, without additional emission reduction measures. The MFR scenario is applied in the future period, considering the maximum achievable emission reduction under existing technological and economic conditions. This implies the implementation of all feasible emission reduction measures, both technically and economically viable, to achieve the greatest reduction in emissions.

**Fig. 7:**
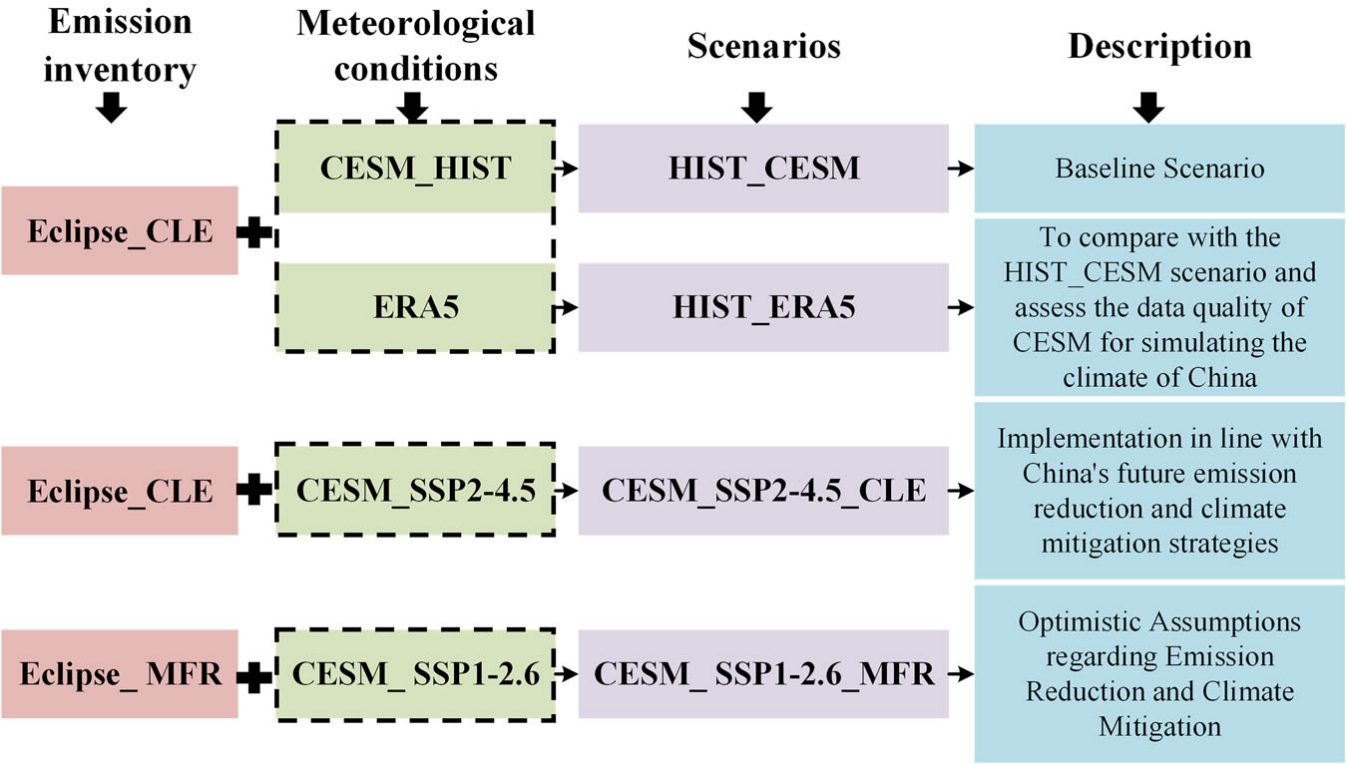
Emission inventory and meteorological conditions in four simulation scenarios.

In this study, four scenarios were designed to investigate the impact of different policy assumptions on PM_2.5_ concentration and health burdens associated with PM_2.5_ exposure in historical and future periods in China (see Fig. 7 for the overview): (1) HIST_CESM: Historical runs (2010–2014) based on CESM_HIST meteorological data and Eclipse_CLE emission assumptions, considered as the baseline scenario. (2) HIST_ERA5: Historical runs (2010–2014) based on ERA5 meteorological data and Eclipse_CLE emission assumptions, used to assess the data quality of CESM for simulating Chinese climate. (3) CESM_SSP2–4.5_CLE: Future runs (2020, 2030, 2040, and 2049) based on CESM_SSP2–4.5 meteorological data and Eclipse_CLE emission assumptions. This scenario is considered to align with the implementation of future emission reduction strategies in China. (4) CESM_SSP1–2.6_MFR: Future runs (2020, 2030, 2040, and 2049) based on CESM_SSP1–2.6 meteorological data and Eclipse_MFR emission assumptions. This scenario is regarded as an optimistic assumption for emission reduction.

### Setup of the DEHM model

A significant number of studies have shown that the DEHM can well capture many features of PM and its precursors’ changes in large-scale space and exhibits good performance^53^. DEHM is also part of the 11 model ensemble in the European Copernicus Atmospheric Service and is countiniously evaluated as part of this service (https://atmosphere.copernicus.eu). This study applies DEHM to predict and assess PM_2.5_ concentrations in a future context. A two-way nested domain^54^ of the DEHM model system is used in this study. A mother domain with a resolution of 150 km × 150 km is employed on a polar stereographic projection, true at 60°N to cover the entire Northern Hemisphere to account for intercontinental transport. The nested domain covered the whole China consisting of 150 × 150 grid cells with a resolution of 50 km × 50 km. Vertically, there are 29 unevenly distributed layers, with the highest level reaching 100 hPa, and the lowest layer is approximately 20 m in height. The time resolution of the DEHM model output is one hour. The meteorological fields are simulated using the WRFv4.1^49^ setup with exactly same domain setup as the DEHM model. The WRF model is hence used to dynamic downscaling the global data. For the historical weather and climate, WRF is driven by ERA5 and CESM_HIST, while CESM_SSP2-4.5 and CESM_SSP1-2.6 is used for the future climate. The gas-phase chemistry module included 66 species, 9 primary particles (including natural particles such as sea salt), and 138 chemical reactions^53^. The SOA are calculated using the volatility basis set (see details in Im et al.^55^). In addition to the anthropogenic emissions, DEHM also includes emissions from biogenic emissions, such as vegetation, sea salt, lightning, soil, etc. The current version of the DEHM model does not include wind-blown, resuspended dust emissions or resuspended road dust.

In this study, the DEHM model uses anthropogenic emissions from Eclipse v6b and biogenic emissions are calculated online using the Model of Emissions of Gases and Aerosols from Nature (MEGAN) (see details in Zare et al.^56^ and Im et al.^55^).

### Anthropogenic emission sources and their sensitivity experiments design

**Fig. 8:**
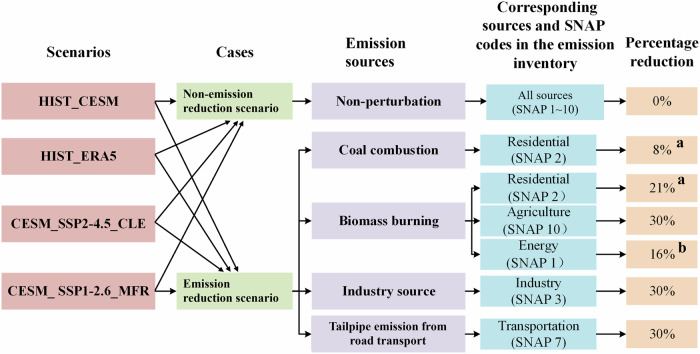
Emission reduction design for anthropogenic emission sources; **a** was obtained from the literature^73^, **b** were obtained from the literature^58,73–79^. SNAP, the acronym for Selected Air Pollution Nomenclature, was developed by the EMEP/EEA (Guidelines for Air Pollutant Emission Inventories) project in synchronization with the IPCC/OECD (Integrated Pollution Prevention and Control) nomenclature for source categories of emitting activities (https://en.eustat.eus/documentos/elem_13173/definicion.html).

The reviews^20,21^ mentioned above show that coal combustion, biomass burning, transportation, and industrial sources are the major anthropogenic emission sources in most parts of China. We conducted sensitivity experiments targeting these four sources to quantitatively assess their contributions to PM_2.5_ concentration and PM_2.5_-related health burdens under different scenarios (see Fig. 8). In this study, the coal combustion refers to coal heating from the residential sector. Biomass burning includes open burning of agricultural biomass, domestic biomass burning for cooking and heating, and biomass burning from biomass power plants and coal-fired power plants, which includes co-firing of biomass and coal. To accelerate carbon reduction in coal-fired power generation, the Chinese government has issued a series of policies supporting and encouraging the coupling of coal and biomass for power generation^57^. This undoubtedly adds complexity to distinguishing between PM_2.5_ emissions from coal combustion and biomass fuel. Furthermore, biomass is a renewable energy source with abundant resources and various forms of utilization. Its effective utilization is expected to help alleviate the global energy shortage and climate change caused by the massive use of fossil fuels. China has taken a series of measures to cope with climate change, the conflict between energy supply and demand, and to protect the ecological environment. Driven by the energy strategy, the scale of biomass utilization and biomass power generation industry in China has been expanding^58^. Considering the aforementioned reasons, we include PM_2.5_ emissions from coal-fired power plants in our analysis. Industry source is primarily from specific industry processes. It is worth noting that in the DEHM model, the transportation source only considers tailpipe emissions from on-road transport, excluding non-tailpipe emissions from vehicles like road dust, brake dust, and tire wear, as well as other transportation sources like ships and airplanes.

These sensitivity experiments were carried out within the four scenarios proposed in Fig. 7, including both the non-perturbation condition (referred to as the NPC case) and perturbation condition (referred to as the PC case), and a total of 50 runs were performed. Under the non-perturbation condition, all aforementioned emission sources were considered. Under the perturbation condition, reduction designs were implemented for emissions from coal combustion, biomass burning, industrial sources, and tailpipe emission from on-road transport. The emission from each individual source is reduced by 30%. The choice of 30% was motivated by the consideration that the perturbation would be large enough to produce a sizeable impact (i.e., more than numerical noise) even at long distances, while small enough to be in the near-linear atmospheric chemistry regime^59,60^. The implementation steps are as follows:

Firstly, in order to estimate PM_2.5_ concentrations from coal combustion for residential heating and biomass burning, this study obtained the percentage contributions of PM_2.5_ emissions from coal combustion for residential heating, domestic biomass burning for cooking and heating to PM_2.5_ emissions of the residential sector, respectively, as well as the percentage contributions of PM_2.5_ emissions from biomass combustion in power plants to the total PM_2.5_ emissions from the power sector, through data collection and calculation.

Then, on this basis, a uniform 30% emission reduction control was applied to the coal combustion for residential heating, biomass burning (including open burning of agricultural biomass, domestic biomass burning for cooking and heating, and biomass burning from biomass power plants and coal-fired power plants), industrial sources, and tailpipe emission from on-road transport studied in this study.

Subsequently, the final percentage reduction was obtained, as shown in Fig. 8. Under the perturbation condition for residential heating coal combustion, 8% emission reduction control was applied to the residential sector; under the perturbation condition for biomass burning, 21%, 30%, and 16% emission reduction control was applied to the residential sector, the agricultural sector, and the energy sector, respectively; under the perturbation condition for the industry source, 30% emission reduction control was applied to the industry sector; and under the perturbation condition for the tailpipe emission from on-road transport, 30% emission reduction control was applied to the tailpipe emission from on-road transport.

Finally, the PM_2.5_ concentration for each sector is calculated using Eq. (1)~(3):

1 $${C}_{P,i}=\frac{{C}_{{NPC},i}-{C}_{{PC},i}}{30 \% }$$

2 $${C}_{P,{primary\; PM}2.5}={C}_{P,{total\; PM}2.5}-{C}_{P,{SIA}}-{C}_{P,{SOA}}$$

3 $${C}_{{secondary}}={C}_{{NPC},{SOA}}+{C}_{{NPC},{SIA}}$$

where, *i* refers to the type of pollutants, i.e., total PM_2.5_, SOA, secondary inorganic aerosols, and primary PM_2.5._ ${C}_{{NPC},i}$ represents concentrations of the pollutant *i* in the NPC case. ${C}_{{PC},i}$ represents concentrations of the pollutant *i* in the PC case. ${C}_{P,i}$ represents concentrations of the pollutant *i* by the specific emission sector P which is perturbed (perturbation sectors P include coal combustion for residential heating, biomass burning, industry sources, tailpipe emission from on-road transport). ${C}_{P,{primary\; PM}2.5}$ represents concentrations of primary PM_2.5_ by the perturbation sectors P. ${C}_{{secondary}}$ represents PM_2.5_ concentrations by the secondary aerosol formation.

### Population and baseline mortality data

Population density data has been taken from the Socioeconomic Data and Applications Center^61^ on a 30 arc-minute spatial resolution, which was subsequently aggregated to the 50 km × 50 km resolution in the nested model domain of DEHM. The dataset includes global population estimates by age and sex for 2010, expressed as counts (number of people per pixel) and densities (number of people per square kilometer), consistent with national censuses and population registers^61^. The dataset is categorized by age into five-year age groups from ages 0–4 to ages 85+^61^. In these data, the age breakdown is only available for 2010. Therefore, the age distribution data for China from World Population Prospects^62^ for 1950–2049 were used to process the population data from the Socioeconomic Data and Applications Center to obtain age distributions for all other years. The age distribution data were initially spaced at 5-year intervals and subsequently processed to the actual years in the EVA by interpolation (see details in Im et al.^14^). Considering that the number of premature deaths attributable to PM_2.5_ as a proportion of the total population is minimal, and to facilitate comparison of the effect of reduced PM_2.5_ emissions on total premature deaths attributable to PM_2.5_, the same population structure is used for the SSP2-4.5 and SSP1-2.6 scenarios. The country-specific baseline mortality for each age group was obtained from Global Burden of Disease^63^. Due to the lack of data on future baseline mortality, this study used 2017 baseline mortality for future scenario projections.

### EVA model and its sensitivity experiments design

The EVA model^64,65^ is based on the impact-pathway chain methodology^66^. As a health impact assessment system, it has been widely applied^14,39,55,64,65,67,68^. Combining calculated air pollutant concentrations with population data, we can calculate human exposure, health effects (morbidity and mortality), and associated external costs^68^. This work focuses on premature mortality. With updates to the EVA model, it is possible to calculate premature deaths due to exposure to air pollutants (including PM_2.5_, SO_2_, NO_2_, and O_3_) using linear ERFs, as well as cause-specific mortality using nonlinear functions based on Burnett et al.^69^.

The linear version of the EVA model (referred to as linear EVA) calculates the health burden using ERFs based on a relative risk of 8.0% for all-cause chronic mortality due to PM_2.5_ based on a review by Chen and Hoek^70^, as recently recommended by the WHO^71^ (denoted as WHO_RR). The nonlinear version of the EVA model (referred to as nonlinear EVA) incorporates nonlinear, location specific functions to estimate cause-specific mortality attributable to PM_2.5_^14^. This version employs the GEMM model to calculate relative risks (RR) using hazard ratio functions^69^. In the present study, the methodology of Burnett et al.^69^ as implemented in Lelieveld et al.^72^ for PM_2.5_-related mortality was followed for estimating the RR as:

4 $${RR}=\exp \left\{\theta \frac{\log \left[\left(\frac{z}{\alpha }\right)+1\right]}{\left(1+\exp \left\{-\frac{\left(z-\mu \right)}{\nu }\right\}\right)}\right\}$$

where *z* = max (0, PM_2.5_–2.4 μg m^−3^), and *θ*, *α*, *μ* and *ν* are variables obtained from Burnett et al.^69^ and *z* is the PM_2.5_ concentration. log is here the natural logarithmic function. The GEMM model adopted in the EVA assumes a cut-off PM_2.5_ concentration of 2.4 μg m^−3^ ^69^. The linear EVA assumes a straight-line relationship between PM_2.5_ concentration and mortality risk, meaning that the risk increases proportionally with increasing PM_2.5_ levels. In contrast, the nonlinear EVA uses more complex that allow for a non-proportional change in risk, with a steeper increase in the impacts at lower concentrations than at higher concentrations^18,19^. For more detailed descriptions of the linear EVA, nonlinear EVA, and GEMM models, please refer to previous publications^14,39,55,64,65,67–69^.

In this study, atmospheric PM_2.5_ concentrations under specific scenarios (CESM_SSP2-4.5_CLE, CESM_SSP1-2.6_MFR) simulated by the DEHM model, including overall PM_2.5_ concentrations or PM_2.5_ concentrations due to specific anthropogenic emission sources (coal combustion for residential heating, biomass burning, tailpipe emission from on-road transport, and industrial sources), as well as projected population data are used as input data to the EVA model to estimate health burdens associated with PM_2.5_ pollution exposures or the effects of specific anthropogenic emission sources on the health burdens associated with them. The study focuses on the following health burdens (as shown in Table 2): the linear EVA was used to estimate premature deaths attributable to PM_2.5_ exposure, including acute deaths due to short-term PM_2.5_ exposure (AD), chronic deaths due to long-term PM_2.5_ exposure (CD), and total deaths due to PM_2.5_ exposure (ADCD = AD + CD). Additionally, the nonlinear EVA model was used to estimate five specific causes of death: lung cancer (LC), chronic obstructive pulmonary disease (COPD), ischemic heart disease (IHD), and stroke – all four regarded as non-communicable diseases (NCD), as well as lower respiratory infections (LRI), together giving a total number of premature deaths attributable to PM_2.5_ exposure.

**Table 2:** The causes of premature deaths attributable to PM_2.5_ exposure explored in this study

Model	Premature deaths
Linear EVA	Acute Deaths (AD)
	Chronic Deaths (CD)
	Total premature deaths (ADCD = AD + CD)
Nonlinear EVA	Lung Cancer (LC)
	Chronic Obstructive Pulmonary Disease (COPD)
	Ischemic Heart Disease (IHD)
	Stroke
	Lower Respiratory Infections (LRI)
	Non-Communicable Diseases (NCD = LC + COPD + IHD+stroke)
	Total premature deaths (=NCD + LRI)

For a detailed description of the method part, i.e., meteorological data and emission inventories, the simulation scenarios, DEHM model setup, and model evaluation, please refer to the previous publication^32^ if the readers are interested.

### Supplementary information

Geographic location of eastern, central, western and north-eastern China (Supplementary Fig. 1), the percentage contributions of anthropogenic emission sources to total PM_2.5_-related premature mortality estimated with the linear EVA from 2010 to 2049 under different scenarios in mainland China (Supplementary Fig. 2), the impact of emission reduction scenarios on the number of premature deaths attributed PM_2.5_ concentration in mainland China from 2010 to 2049 (Supplementary Table 1) and percentage of PM_2.5_-related premature deaths in China by region in relation to total PM_2.5_-related premature deaths in China (Supplementary Table 2) are provided in the Supplementary Materials file.

## Supplementary information

Supplementary information

## Data Availability

All CMIP6_CESM results can be obtained at https://esgf-node.llnl.gov/search/cmip6/. The emission inventory can be obtained at https://previous.iiasa.ac.at/web/home/research/researchPrograms/air/Global_emissions.html. Population density data can be obtained at https://sedac.ciesin.columbia.edu/data/collection/gpw-v4/sets/browse. Other data are available from the corresponding author upon request.
