# Supplementary material for: The recent and future PM2.5-related health burden in China apportioned by emission source
Source: NPJ Clean Air. 2025 Apr 28;1(1):7. doi: 10.1038/s44407-025-00006-9 (PMC12037407; doi:10.1038/s44407-025-00006-9)
Supplement: Supplementary file 1 — Supplementary information [file 44407_2025_6_MOESM1_ESM.pdf]

Supplemantrary Material

The recent and future $PM_{2.5}$-related health burden in China apportioned by emission source

Jiemei Liu[1,2], Jørgen Brandt[2], Jesper H. Christensen[2], Zhuyun Ye[2], Tingsen Chen[3], Shikui Dong[1], Camilla Geels[2], Yuan Yuan[1,4*], Athanasios Nenes[5,6] and Ulas Im[2,*]

[1] Key Laboratory of Aerospace Thermophysics, Ministry of Industry and Information Technology, Harbin Institute of Technology, 92 West Dazhi Street, Harbin 150001, China

[2] Aarhus University, Department of Environmental Science/Interdisciplinary Centre for Climate Change, Frederiksborgvej 399, Roskilde, Denmark

[3] School of Mechanical Engineering, Beijing Institute of Technology, Beijing 100081, China

[4] School of Urban Construction, Wuhan University of Science and Technology, No. 2, West Huangjiahu Road, Hongshan District, Wuhan 430065, China

[5] Laboratory of Atmospheric Processes and Their Impacts, Ecole Polytechnique Fédérale de Lausanne (EPFL), Lausanne, Switzerland

[6] Center for the Study of Air Quality and Climate Change, Foundation for Research and Technology Hellas (FORTH), Thessaloniki, Greece

*Correspondence: Yuan Yuan (yuanyuan83@hit.edu.cn) and Ulas Im (ulas@envs.au.dk)

Captions of Supplementary Tables

Supplementary Figure 1. Geographic location of eastern, central, western and north-eastern China

Supplementary Figure 2. The percentage contributions of anthropogenic emission sources to total $PM_{2.5}$-related premature mortality estimated with the linear EVA from 2010 to 2049 under different scenarios in mainland China

Supplementary Table 1. The impact of emission reduction scenarios on the number of premature deaths attributed $PM_{2.5}$ concentration in mainland China from 2010 to 2049

Supplementary Table 2. Percentage of $PM_{2.5}$-related premature deaths in China by region in relation to total $PM_{2.5}$-related premature deaths in China

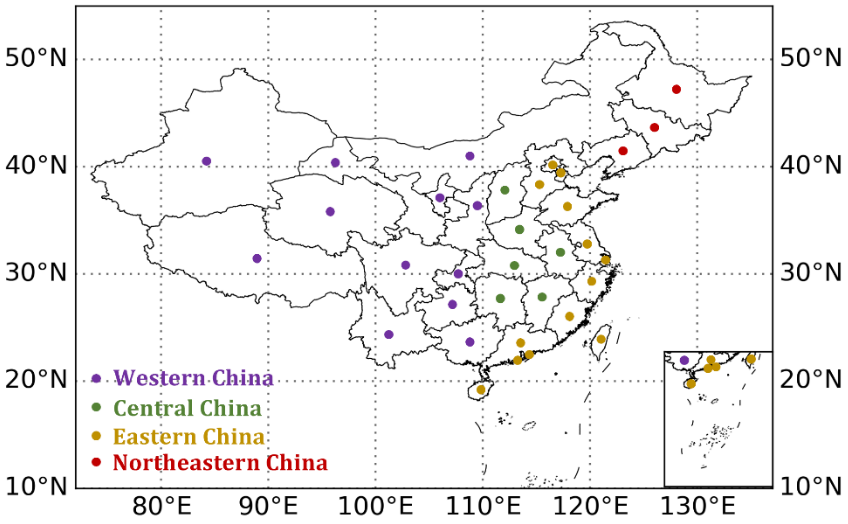

Supplementary Figure 1. Geographic location of eastern, central, western and north-eastern China. Green dots represent the central region of China, comprising Shanxi, Anhui, Jiangxi, Henan, Hubei, and Hunan provinces. Yellow dots represent the eastern region of China, comprising Beijing, Tianjin, Hebei, Shanghai, Jiangsu, Zhejiang, Fujian, Shandong, Guangdong, Hainan, Hong Kong, Macao, and Taiwan; It should be noted that the eastern region in this study includes Hong Kong, Macao and Taiwan. Purple dots represent the western region of China, comprising twelve provinces (autonomous regions and municipalities): Inner Mongolia, Guangxi, Chongqing, Sichuan, Guizhou, Yunnan, Tibet, Shaanxi, Gansu, Qinghai, Ningxia and Xinjiang. Red dots represent the northeastern region of China, comprising Liaoning, Jilin and Heilongjiang provinces.

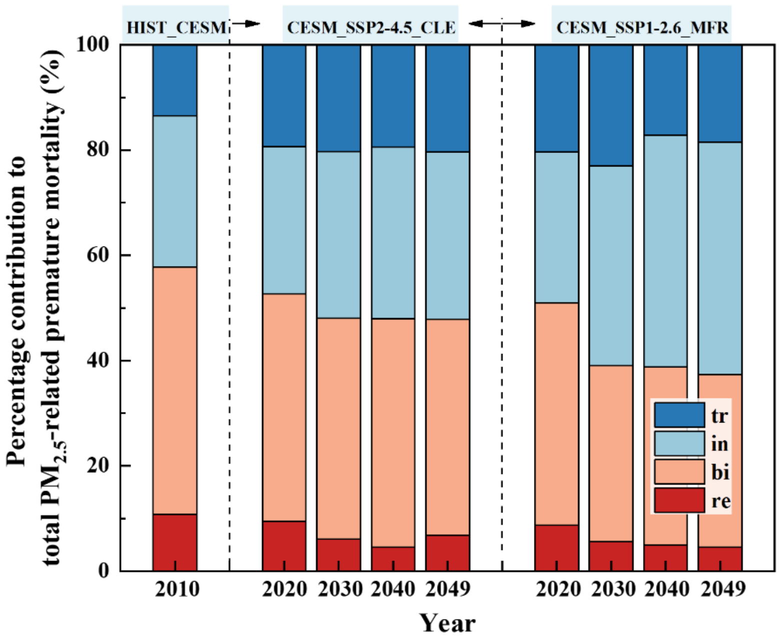

Supplementary Figure 2. The percentage contributions of anthropogenic emission sources to total $PM_{2.5}$-related premature mortality estimated with the linear EVA (RR=8.0%, recorded as WHO_RR) from 2010 to 2049 under different scenarios in mainland China. Anthropogenic emission sources include coal combustion for residential heating (re), biomass burning (bi), industry (in), and tailpipe emission from on-road transport (tr). Three zones from left to right represent HIST_CESM, CESM_SSP2-4.5_CLE, and CESM_SSP1-2.6_MFR scenarios. Dark blue, light blue, orange, and red rectangles represent the percentage contributions of tailpipe emission from on-road transport (tr), industry (in), biomass burning (bi), and coal combustion for residential heating (re) to total $PM_{2.5}$-related premature deaths, respectively.

Supplementary Table 1. The impact of emission reduction scenarios on the number of premature deaths attributed $PM_{2.5}$ concentration in mainland China from 2010 to 2049.

Item	Percentage change in premature deaths/$PM_{2.5}$	2020	2030	2040	2049
WHO_RR	AD	0.4%	-52.3%	-62.3%	-65.3%
	CD	0.3%	-52.2%	-62.0%	-65.0%
	Total deaths	0.3%	-52.2%	-62.0%	-65.0%
Non-linear	LC	0.7%	-46.3%	-57.6%	-61.4%
	COPD	0.8%	-45.4%	-56.7%	-60.6%
	IHD	0.6%	-37.5%	-47.6%	-51.0%
	stroke	0.9%	-51.8%	-62.8%	-66.5%
	LRI	0.7%	-45.8%	-60.3%	-65.1%
	Total deaths	0.5%	-39.8%	-49.7%	-53.0%
Air pollutant	$PM_{2.5}$	0.7%	53.5%	62.5%	66.1%

*Note: AD, CD, LC, COPD, IHD, and LRI correspond to acute deaths due to short-term exposures, chronic deaths due to long-term exposures, lung cancer, chronic obstructive pulmonary disease, ischemic heart disease, and lower respiratory infections respectively; percentage change refers to the percentage change in premature mortality/$PM_{2.5}$ concentration under CESM_SSP1-2.6_MFR relative to CESM_SSP2-4.5_CLE

Supplementary Table 2. Percentage of $PM_{2.5}$-related premature deaths in China by region in relation to total $PM_{2.5}$-related premature deaths in China

Model	Scenarios	Year	Eastern China	Central China	Western China	North-eastern China	Central and eastern China
Linear EVA	HIST_CESM	2010	40.4%	31.2%	21.7%	6.8%	71.6%
	CESM_SSP2-4.5_CLE	2020	41.5%	30.4%	20.8%	7.3%	71.9%
		2030	62.3%	15.1%	16.4%	6.2%	77.3%
		2040	63.1%	14.9%	15.4%	6.6%	78.0%
		2049	62.2%	15.2%	15.6%	6.9%	77.5%
	CESM_SSP1-2.6_MFR	2020	41.6%	31.2%	20.6%	6.6%	72.8%
		2030	63.2%	14.0%	16.1%	6.7%	77.2%
		2040	64.6%	13.8%	15.1%	6.6%	78.3%
		2049	65.1%	13.1%	14.5%	7.2%	78.2%
Non-linear EVA	HIST_CESM	2010	36.7%	31.4%	25.8%	6.1%	68.1%
	CESM_SSP2-4.5_CLE	2020	37.2%	31.1%	25.4%	6.3%	68.3%
		2030	56.8%	15.4%	21.3%	6.5%	72.2%
		2040	57.4%	15.6%	20.3%	6.7%	73.0%
		2049	56.9%	15.9%	20.2%	7.0%	72.8%
	CESM_SSP1-2.6_MFR	2020	37.3%	31.6%	25.2%	5.9%	69.0%
		2030	59.8%	15.5%	18.0%	6.7%	75.3%
		2040	62.2%	16.0%	15.8%	6.0%	78.1%
		2049	62.9%	15.7%	14.9%	6.6%	78.6%
